# Comparison of Two Transmission Electron Microscopy Methods to Visualize Drug-Induced Alterations of Gram-Negative Bacterial Morphology

**DOI:** 10.3390/antibiotics10030307

**Published:** 2021-03-17

**Authors:** Hang Thi Nguyen, Lisa A. O’Donovan, Henrietta Venter, Cecilia C. Russell, Adam McCluskey, Stephen W. Page, Darren J. Trott, Abiodun D. Ogunniyi

**Affiliations:** 1 Australia Centre for Antimicrobial Resistance Ecology, School of Animal and Veterinary Sciences, Roseworthy Campus, The University of Adelaide, Roseworthy, SA 5371, Australia; hang.t.nguyen@adelaide.edu.au; 2 Department of Pharmacology, Toxicology, Internal Medicine and Diagnostics, Faculty of Veterinary Medicine, Vietnam National University of Agriculture, Hanoi 100000, Vietnam; 3 ARC Centre of Excellence in Plant Energy Biology, School of Agriculture Food & Wine, Waite Campus, The University of Adelaide, Urrbrae, SA 5064, Australia; lisa.odonovan@adelaide.edu.au; 4 Health and Biomedical Innovation, Clinical and Health Sciences, University of South Australia, Adelaide, SA 5000, Australia; rietie.venter@unisa.edu.au; 5 Chemistry School of Environmental and Life Sciences, University of Newcastle, Callaghan, NSW 2308, Australia; cecilia.russell@newcastle.edu.au (C.C.R.); adam.mccluskey@newcastle.edu.au (A.M.); 6 Neoculi Pty Ltd., Burwood, VIC 3125, Australia; swp@advet.com.au

**Keywords:** transmission electron microscopy, bacterial cell wall, bacterial membrane, Gram-negative bacteria, colistin, drug interaction, Tokuyasu cryo-ultramicrotomy

## Abstract

In this study, we optimized and compared different transmission electron microscopy (TEM) methods to visualize changes to Gram-negative bacterial morphology induced by treatment with a robenidine analogue (NCL195) and colistin combination. Aldehyde-fixed bacterial cells (untreated, treated with colistin or NCL195 + colistin) were prepared using conventional TEM methods and compared with ultrathin Tokuyasu cryo-sections. The results of this study indicate superiority of ultrathin cryo-sections in visualizing the membrane ultrastructure of *Escherichia coli* and *Pseudomonas aeruginosa*, with a clear delineation of the outer and inner membrane as well as the peptidoglycan layer. We suggest that the use of ultrathin cryo-sectioning can be used to better visualize and understand drug interaction mechanisms on the bacterial cell membrane.

## 1. Introduction

Gram-negative bacterial pathogens exhibit high-level resistance to most classes of antibiotics due to the presence of an impermeable outer membrane [1,2]. Polymyxins are considered as last-line agents for the treatment of Gram-negative infections due to their unique mechanism of action targeting the outer membrane [3,4,5,6]. However, polymyxins are highly nephrotoxic and neurotoxic agents if high doses are used [7,8], resulting in a narrow therapeutic window for Gram-negative infections. The usage of polymyxins in combination with other agents is being considered as a strategy for overcoming reduced polymyxin susceptibility and toxicity without increasing polymyxin exposure [3,9]. The mechanism of beneficial combination treatment is proposed to involve complete integration of polymyxins into the outer membrane causing disorganization and neutralization of cell surface charge and consequently loss of envelope barrier function. Subsequently, the affected outer membrane is hypothesized to transiently open, allowing entry of the second antibiotic and interaction with otherwise inaccessible drug target sites [2,10,11,12,13].

**Figure 1:**
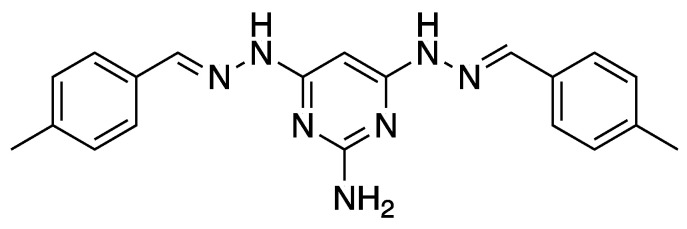
Chemical structure of novel pyrimidine NCL195, 4,6-bis(2-((*E*)-4-methylbenzylidene)hydrazinyl)pyrimidin-2-amine.

Our ongoing studies have indicated potential therapeutic options using the novel pyrimidine NCL195, 4,6-bis(2-((*E*)-4-methylbenzylidene)hydrazineyl)pyrimidin-2-amine (Figure 1) combined with subinhibitory concentrations of polymyxin B (PMB) or colistin against Gram-negative infections [10,11]. We showed synergistic activity of the NCL195-PMB or NCL195-colistin combination against clinical Gram-negative bacterial pathogens, with MICs for NCL195 ranging from 0.25–4 µg/mL for *Acinetobacter baumannii*, *Escherichia coli*, *Klebsiella pneumoniae* and *Pseudomonas aeruginosa*, whereas NCL195 alone had no activity.

For decades, transmission electron microscopy (TEM) has been a valuable research tool in microbiology for high-resolution structural studies of bacteria and their components [14,15]. TEM was applied to study the effect of drug treatment on both Gram-negative and Gram-positive bacteria [16,17]. We have also used TEM to study NCL195-colistin interactions on the Gram-negative cell membrane [10]. During the investigation, the stability of Gram-negative bacterial cell morphology was affected by several factors, including buffer conditions, selected fixatives, type of resin and the embedding method. Cryo-EM and Tokuyasu cryo-ultramicrotomy have been shown to offer some advantages over conventional TEM for investigating bacterial ultrastructure, including better resolution, artifact reduction, clearer visualization of bacterial cytoskeleton and better preservation of bacterial structural integrity [18,19,20,21,22,23]. Therefore, determining the most effective technique to accurately visualize and elucidate drug interactions on bacteria is essential. The objective of the present investigation was to compare two sample preparation methods for TEM (conventional resin embedding and Tokuyasu cryo-ultramicrotomy) to visualize the morphological changes occurring on the cell membrane of *E. coli* and *P. aeruginosa* after exposure to NCL195 alone, colistin alone or NCL195-colistin combination.

## 2. Materials and Methods

### 2.1. Antibiotics and Chemicals

NCL195, a novel pyrimidine compound [24,25] (Figure 1), was synthesized at the University of Newcastle. The compound was stored in a sealed container in the dark at 4 °C at the Infectious Diseases Laboratory, Roseworthy campus, The University of Adelaide. Colistin sulphate, kanamycin and tetracycline were purchased from Sigma-Aldrich (Australia). Stock solutions containing 25.6 mg/mL of each compound (NCL195 dissolved in DMSO, colistin and kanamycin dissolved in water and tetracycline dissolved in 70% of ethanol) were stored in 1 mL aliquots at −20 °C away from direct light. Ruthenium red, L-lysine acetate and sucrose were purchased from Sigma-Aldrich, Australia, and dissolved in water to the appropriate concentrations. Fixatives and cacodylate buffer were provided by Adelaide Microscopy, The University of Adelaide, Adelaide, South Australia, Australia.

### 2.2. Bacterial Strains and Growth Conditions

Bioluminescent *E. coli* Xen14 (derived from the parental strain *E. coli* WS2572*)* and bioluminescent *P. aeruginosa* Xen41 (derived from the parental strain PAO1) were purchased from PerkinElmer Inc. (Waltham, MA, USA). *E. coli* Xen14 was grown on horse blood agar (HBA) containing 30 µg/mL kanamycin and *P. aeruginosa* Xen41 was grown in HBA containing 60 µg/mL tetracycline overnight at 37 °C in normal air for selection. A single colony was taken from the overnight growth, suspended in 10 mL Luria-Bertani (LB) broth, Miller (Becton Dickinson, Sparks, MD, USA) in a 50 mL flask and incubated at 37 °C under continuous agitation in an orbital shaker at 150 rpm. The overnight bacterial culture was diluted 1:30 in 40 mL LB broth in 50 mL flask of LB broth and then incubated again at 37 °C under continuous agitation until *A*_600nm_ = 0.1 or 0.5.

### 2.3. Transmission Electron Microscopy

#### 2.3.1. Xen14 Processing for TEM

Xen14 was prepared in five different ways (Table 1) to minimize factors that may affect the quality of TEM images. The Xen14 cells were cultured as described above, and then harvested by centrifugation at 2900× *g* for 5 min at 4 °C to avoid cell damage. The cells were initially resuspended in either cacodylate buffer (pH 7.0) or phosphate-buffered saline (PBS; pH 7.0) and centrifuged twice for 5 min at 2900× *g*. Thereafter, cell pellets were fixed overnight in fixative containing 3.0% formaldehyde, 0.035% glutaraldehyde, 4% sucrose in cacodylate buffer (Procedure 1); fixative containing 4.0% formaldehyde, 1.25% glutaraldehyde, 4% sucrose in PBS buffer (Procedure 2); fixative containing 4.0% formaldehyde, 1.25% glutaraldehyde in cacodylate buffer without sucrose supplementation (Procedure 3); fixative containing 4.0% formaldehyde, 1.25% glutaraldehyde, 4% sucrose and 0.01 M CaCl_2_ in cacodylate buffer (Procedures 4 and 5), as detailed in Table 1. The fixed cells were then washed in the corresponding buffer as described above, and post-fixed in 1% osmium tetroxide in cacodylate buffer or PBS containing 0.075% ruthenium red for 1 h, and subsequently washed as described above. Cells were then dehydrated in graded series of ethanol (50%, 70%, 90%, 2× each for 10 min and 100%, 3× for 15 min). Thereafter, the cells were infiltrated for 1 h each in propylene oxide: Epon-Araldite resin (50:50 ratio; Procedures 2, 3 and 5) or 100% ethanol: LR-White resin (50:50 ratio; Procedures 1 and 4). Samples were incubated in 100% Epon-Araldite resin (Procedures 2, 3 and 5) or LR-White resin (Procedures 1 and 4) overnight, followed by two resin changes 5 h apart the following day. Subsequently, the cells were polymerized in fresh Epon-Araldite resin or LR-White resin at 70 °C or 58 °C, respectively, for 48 h.

#### 2.3.2. Xen41 Processing for TEM

Xen41 cells were prepared essentially as described for Xen14, then processed for TEM using Procedure 4 (Table 1) with either 1 h fixation or overnight fixation followed by post fixation in 1% osmium tetroxide for 1.5 h on ice.

Sections of Xen14 and Xen41 embedded in resin were cut to 1 μm using a glass knife, stained with 1% toluidine blue containing 1% borax and viewed under a light microscope at 400× magnification to identify stained bacteria. Ultrathin sections were then cut to 90 nm with an ultramicrotome EM-UC6 (Leica) using a diamond knife (Diatome) and placed on 200-mesh copper EM grids (Proscitech). Sections were sequentially stained with uranyl acetate (4% in distilled H_2_O) and Reynolds lead citrate for 10 min each, with three washes in distilled water in-between each stain. Sections were then viewed on a Tecnai G2 Spirit (FEI Company, Hillsboro, OR, USA) Transmission Electron Microscope operated at 100 KV at Adelaide Microscopy, The University of Adelaide.

#### 2.3.3. Cryo-Ultramicrotomy

Xen14 and Xen41 cells were prepared as described above before being fixed in 1 mL cacodylate buffer containing 4.0% formaldehyde, 1.25% glutaraldehyde, 0.01 M CaCl_2_, 4% sucrose and 0.075% ruthenium red, 0.075% L-lysine acetate (to stabilize the peptidoglycan layer and aid in locating the bacteria during sectioning) [26,27]. Samples were then stored at 4 °C until processing for cryo-ultramicrotomy. Thereafter, cells were washed twice in buffer and embedded in 12% gelatin. Small gelatin blocks containing bacteria (<1 mm^3^) were cut and infiltrated with 2.3 M sucrose in phosphate buffer overnight at 4 °C with gentle rocking. Blocks were stored in 2.3 M sucrose at 4 °C prior to sectioning. Blocks were transferred to aluminum sectioning pins (Leica) and quickly plunge-frozen in liquid nitrogen. Thin cryo-sections (80 nm) were cut at −100 °C with an EM-UC6/FC7 cryo-ultramicrotome (Leica) using a cryo-diamond knife (Diatome). Cryo-sections were removed from the knife with 2.3 M sucrose using a wire loop and transferred to formvar/carbon-coated, plasma-cleaned 200-mesh copper EM grids. Grids were stored in an airtight container on sucrose droplets at 4 °C. To stain, grids were floated face down on 2% gelatin for 30 min at 37 °C before washing in PBS (3 × 2 min) and staining with 2% uranyloxalate acetate pH7 (5 min, 22 °C) and methyl cellulose–uranyl acetate (pH 4) on ice (10 min). Grids were looped out, drained and allowed to dry. Samples were imaged with a Tecnai G2 Spirit electron microscope (FEI Company) operated at 100 kV at Adelaide Microscopy, The University of Adelaide.

### 2.4. Treated Samples Processing for TEM and Cryo-Ultramicrotomy

To determine the optimal conditions to observe NCL195-colistin interaction on Gram-negative membranes, Xen14 cells were initially grown until *A*_600nm_ = 0.1 (early logarithmic phase) and 0.5 (mid logarithmic phase) and then treated with colistin at 0.5 µg/mL for 1 h. Subsequently, Xen14 cells grown to *A*_600nm_ = 0.1 were chosen for further analysis and were incubated with 0.5 µg/mL colistin for 2 h and 4 h to determine optimal treatment time.

To determine NCL195–colistin interaction on the cell membrane, bacterial samples were treated as follows: For Xen14, (i) no treatment, (ii) NCL195 alone (2 µg/mL); (iii), colistin alone (0.125 µg/mL); (iv), colistin alone (0.25 µg/mL); (v), colistin (0.25 µg/mL) + NCL195 (2 µg/mL) combination. For Xen 41, cells were washed twice in cacodylate buffer and resuspended in LB broth to *A*_600nm_ = 0.1. Aliquots were then treated as follows: (i) no treatment, (ii) NCL195 alone (2 µg/mL); (iii) colistin alone (1 µg/mL); (iv) colistin (1 µg/mL) + NCL195 (2 µg/mL) combination. Aliquots of Xen14 and Xen41 were then incubated for 1 h at 37 °C under continuous agitation in a shaker at 150 rpm. During the treatment time, each sample was manually mixed every 10 min to ensure adequate antibiotic contact with the cells. Following treatment, Xen14 cells were washed in buffer and then fixed overnight according to Procedures 4 or 5 (Table 1), whereas treated Xen41 cells were initially fixed for 1 h then washed twice, fixed again for 1.5 h and subjected to processing as described in Procedure 4 (Table 1). For cryo-ultramicrotomy, both Xen14- and Xen41-treated cells were fixed using Procedure 6 (Table 1) and stored at 4 °C until processing as described above.

## 3. Results and Discussion

### 3.1. Bacterial Cell Morphology Is Affected by the Fixative Used, Buffer Conditions and the Embedding Method

In this work, we sought to determine the most effective technique to accurately visualize drug interactions on the bacterial membrane as part of our on-going research aimed at gaining a better understanding of the complex interactions between membrane-active drugs and the consequent morphological changes occurring on the bacterial surface. To accomplish this, we compared two sample preparation methods for TEM (conventional resin embedding and cryo-ultramicrotomy) to visualize the cell membrane of *E. coli* and *P. aeruginosa* after exposure to NCL195 alone, colistin alone or NCL195-colistin combination. For this study, we examined the morphological changes to bacterial cells exposed to the test drugs using bioluminescent derivatives of *E. coli* and *P. aeruginosa* used routinely in our real-time in vivo assessments of drug efficacy. We initially used Xen14 cells to optimize the best TEM protocol for observing NCL195-colistin interaction on Gram-negative membranes. We found that several factors affected the morphology of the bacterial cells.

**Figure 2:**
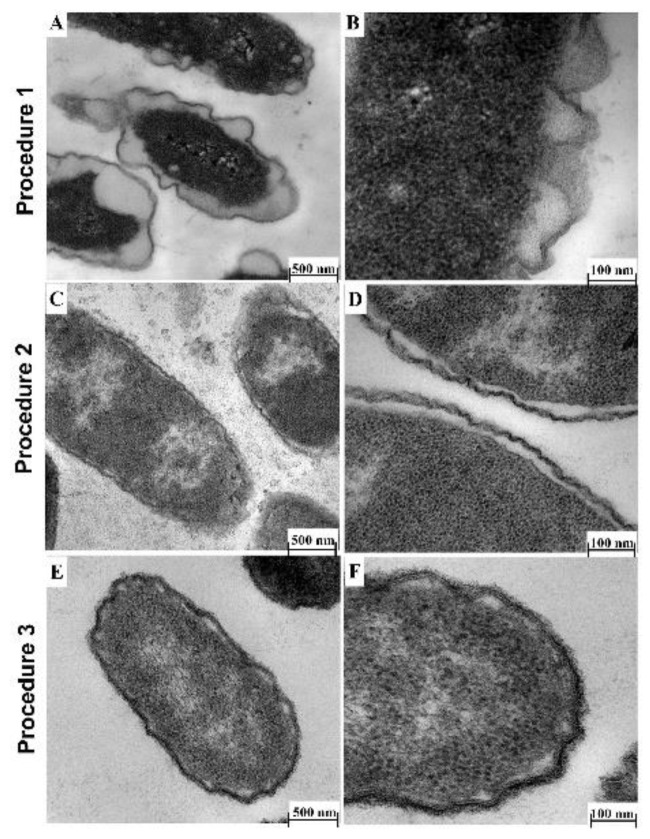
Effect of fixatives and buffer conditions on morphology of Xen14 cells by TEM. (**A**,**B**) TEM (Procedure 1) using fixatives containing 3.0% formaldehyde, 0.035% glutaraldehyde and 4% sucrose in LR-White resin; (**C**,**D**) TEM (Procedure 2) using PBS buffer containing 4.0% formaldehyde, 1.25% glutaraldehyde, 4% sucrose and embedded in Epon resin; (**E**,**F**) TEM (Procedure 3) using cacodylate buffer 4.0% formaldehyde, 1.25% glutaraldehyde and Epon resin embedding without sucrose supplementation.

**Figure 3:**
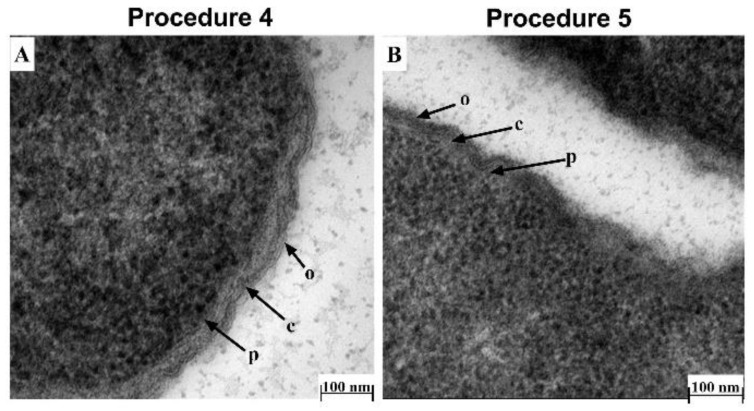
Effect of resin embedding on the morphology of Xen14 cells under optimized TEM conditions. TEM comparison of Xen14 morphology using cacodylate buffer and fixative containing 4.0% formaldehyde, 1.25% glutaraldehyde, 4% sucrose and 0.01 M CaCl_2_ and following different embedding techniques: (**A**) LR-White resin embedding; and (**B**) Epon resin embedding. Cells clearly showed outer membrane (o), cell wall (c) and plasma membrane (p).

- Fixative: Fixative containing 3.0% formaldehyde, 0.035% glutaraldehyde, 4% sucrose and 0.075% ruthenium red, 0.075% L-lysine acetate (Procedure 1) caused shrinkage of the bacterial cell as well as detachment and perturbation of the cell membrane (Figure 2A,B). Therefore, the low concentration of formaldehyde and glutaraldehyde used in this procedure was not high enough to preserve cell membrane structure and could have affected the cell size. To circumvent this, Li*,* et al. [28] described a 4.0% formaldehyde solution in fixative as optimal for preservation of bacterial cell size, and our result supports this observation.
- Buffer: We also found that the type of buffer used resulted in altered cell membrane morphology. PBS buffer caused detachment of the cell membrane (Figure 2C,D; Procedure 2), a similar observation as those described by others [14,29]. Furthermore, the addition of sucrose to the buffer improved preservation of cell morphology, as the cell membrane appeared brittle if sucrose was omitted from the fixative (Figure 2E,F; Procedure 3), in agreement with a previous study [30].
- Embedding method: Following from the optimized fixative and buffer conditions above (Procedure 2), we observed that a TEM protocol using cacodylate buffer with fixative containing 4.0% formaldehyde, 1.25% glutaraldehyde, 0.075% ruthenium red, 0.075% L-lysine acetate, and 4% sucrose followed by embedding in LR-White resin (Procedure 4) provided the best delineation of the outer membrane, cell wall and inner membrane, with no wavy, detached or shrunk membranes (Figure 3A). This protocol is similar in some respects to that described by Voget et al. [14], but differs in the buffer and fixative composition, treatment time and embedding method. The use of Epon-Araldite resin (Procedure 5) did not appear to make a difference to the overall TEM result (Figure 3A vs. Figure 3B). However, given our findings, we suggest using LR-White resin due to its ease of use during TEM processing.

**Figure 4:**
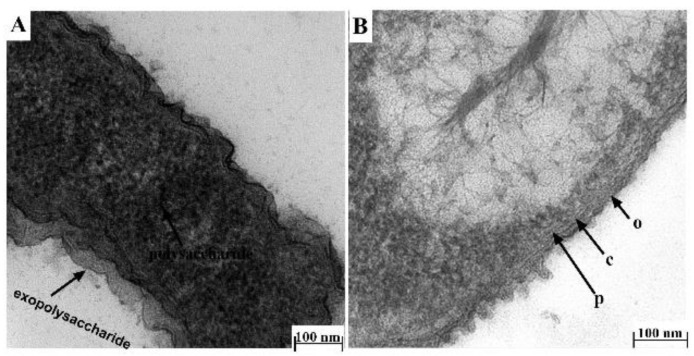
Effect of fixation duration and growth medium on the morphology of Xen41 cells. TEM comparison of Xen41 morphology using cacodylate buffer and fixative containing 4.0% formaldehyde, 1.25% glutaraldehyde, 4% sucrose and 0.01 M CaCl_2_ and following different fixation times and growth in different media: (**A**) Xen41 was cultured overnight in LB broth, washed and fixed overnight and then processed according to Procedure 4; (**B**) Xen41 was grown overnight on horse blood agar initially, washed and fixed for 1 h, washed, and fixed again for an additional 1.5 h and then processed according to Procedure 4.

Having optimized the best TEM protocol for visualizing Xen14 morphology (Procedure 4), this was then applied to *P. aeruginosa* Xen41. However, due to the production of exopolysaccharide by *P. aeruginosa* [15], which may prevent access of fixatives to bacterial cell membrane, Xen41 was grown overnight on horse blood agar to reduce the amount of polysaccharide produced. Cells were washed twice in PBS buffer as described before fixation. Overnight fixation resulted in darker images which masked cell membranes/walls (Figure 4A). Therefore, the procedure was modified by first fixing cells for 1 h, followed by washing in PBS buffer and a subsequent fixation step for 1.5 h before continuing the process as described in Procedure 4. Using this method, the inner membrane, cell wall and outer membrane could clearly be observed under TEM (Figure 4B).

### 3.2. Bacterial Cell Morphology Is Affected by Cell Density and Exposure Time to Drugs

**Figure 5:**
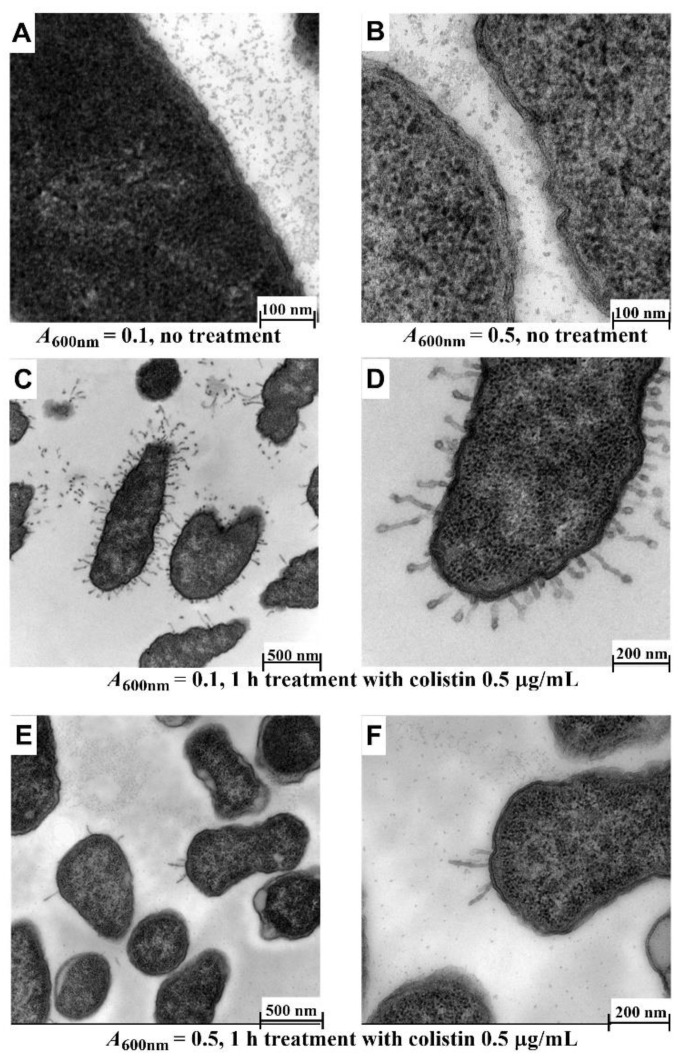
Effect of cell density on morphology of Xen14 cells after treatment with colistin at 0.5 µg/mL for 1 h. (**A**,**B**) Untreated cells; (**C**,**D**) cells grown to *A*_600nm_ = 0.1; (**E**,**F**) cells grown to *A*_600nm_ = 0.5. Cacodylate buffer and fixative containing 4.0% formaldehyde, 1.25% glutaraldehyde, 4% sucrose and 0.01 M CaCl_2_ was used and embedded in Epon resin.

It is known that colistin interacts with the lipopolysaccharide on the surface of Gram-negative bacteria and then across the outer membrane via the self-promoted uptake pathway, resulting in disruption of the normal barrier property of the outer membrane [2,31]. Subsequently, the outer membrane is hypothesized to transiently open thereby allowing passage of NCL195 into the cell to the drug target site(s), likely to be located on the plasma membrane, as we described recently [11]. Based on this hypothesis, we initially determined the optimal time point of colistin treatment that would result in the disruption of the outer membrane using two growth stages of Xen14 (*A*_600nm_ = 0.1 or *A*_600nm_ = 0.5). For this initial analysis, the Xen14 cells were treated with colistin at 0.5 µg/mL for 1 h. Significant morphological changes were observed following 1 h incubation in colistin at *A*_600nm_ = 0.1. Compared to untreated cells (Figure 5A,B), the majority of cells showed a swollen envelope morphology with tubular and fimbria-like radiant appendages; different layers of membrane structure could also be distinguished (Figure 5C,D) under these conditions. However, treatment at *A*_600nm_ = 0.5 showed less effect (Figure 5E,F). Therefore, *A*_600nm_ = 0.1 was used for subsequent experiments as it gave better results.

**Figure 6:**
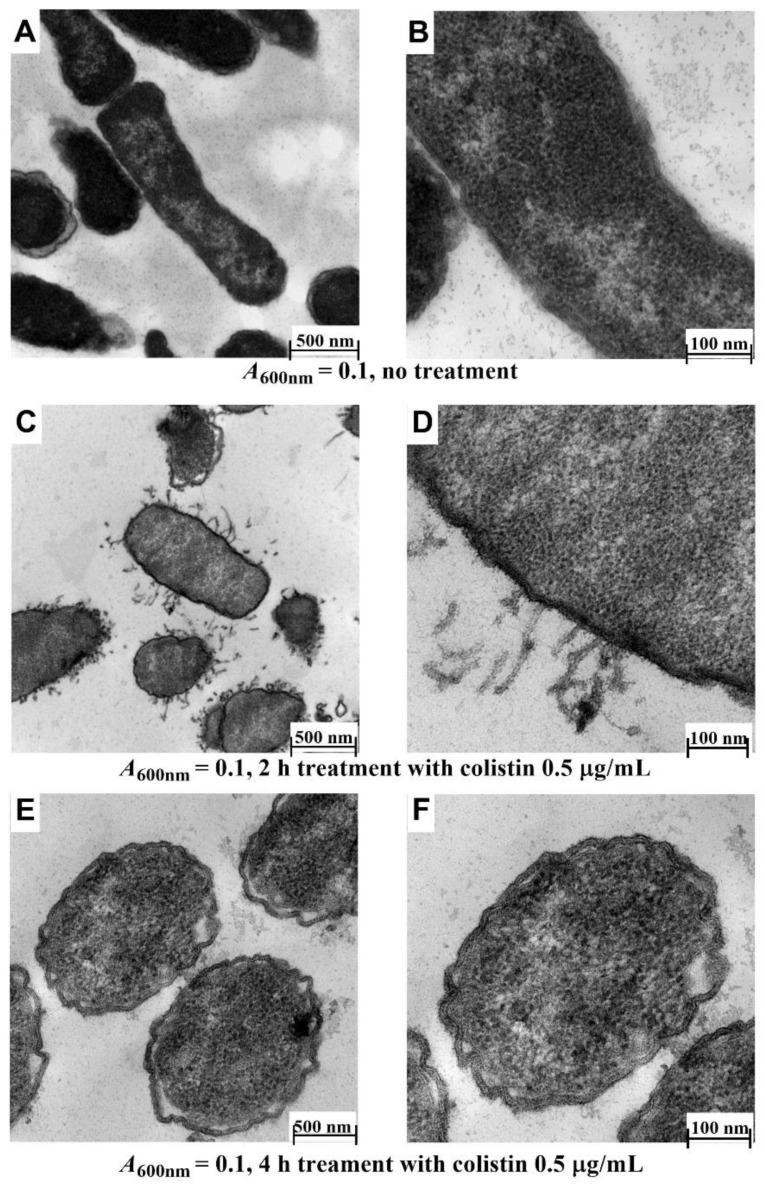
Effect of exposure time on morphology of Xen14. Cells were grown to *A*_600nm_ = 0.1 and then treated with colistin at 0.5 µg/mL. (**A**,**B**) untreated cells; (**C**,**D**) cells treated for 2 h; (**E**,**F**), cells treated for 4 h. Cacodylate buffer and fixative containing 4.0% formaldehyde, 1.25% glutaraldehyde, 4% sucrose and 0.01 M CaCl_2_ was used and embedded in Epon resin.

The effect of 2 h and 4 h colistin treatments on Xen14 cells grown to *A*_600nm_ = 0.1 was also investigated. Compared to untreated cells (6A,B), the majority of the tubular and fimbria-like radiant appendages were broken and had disappeared, although layered membrane structure could still be observed (Figure 6C,D) after 2 h treatment. Following 4 h treatment, all tubular and fimbria-like radiant appendages had disappeared, membrane layers could not be distinguished, and shrinkage of cell contents and detached/wavy membrane structures were observed (Figure 6E,F). These results are similar to those reported previously for polymyxin B [14], a drug with a similar mechanism of action to colistin [32].

### 3.3. Comparison of TEM and Cryo-Ultramicrotomy for Visualizing NCL195-Colistin Interaction on Cell Membrane

**Figure 7:**
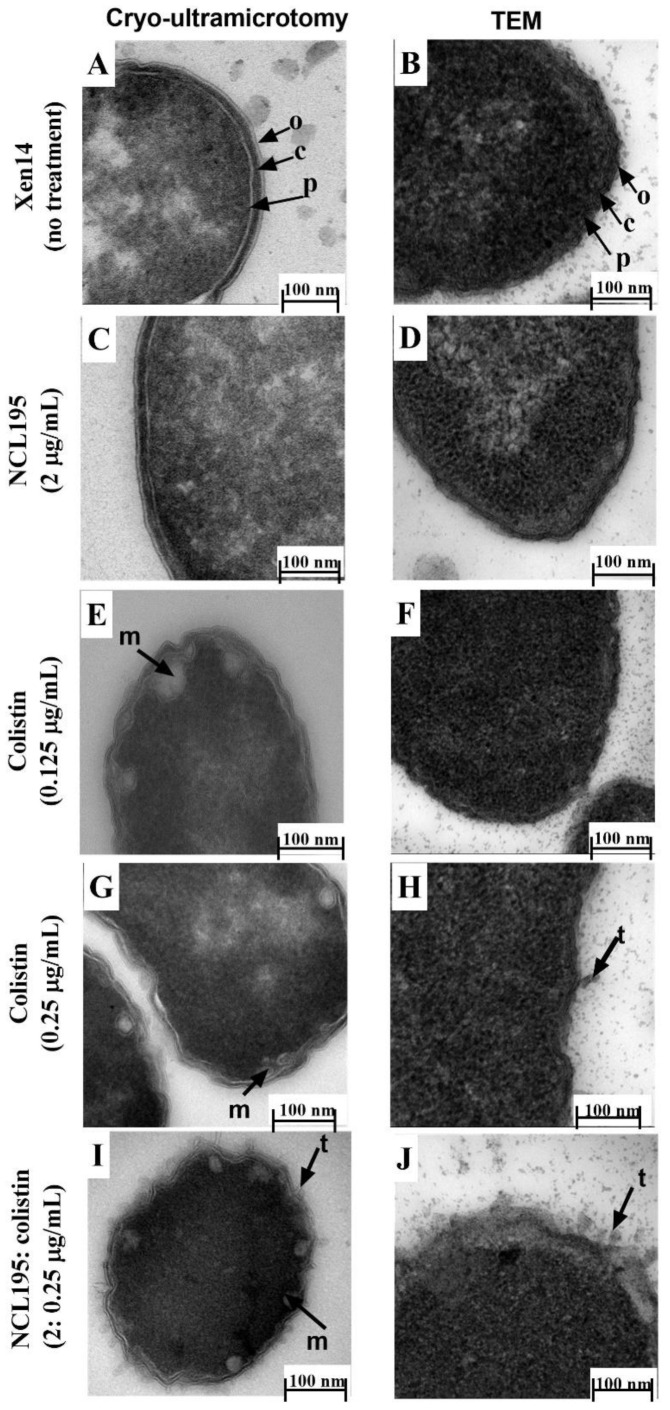
Comparison of Xen14 cells with and without antibiotic treatment and processed by Tokuyasu cryo-ultramicrotomy vs. conventional TEM. Approximately 5 × 10^8^ cells were treated for 1 h; Xen14 cells. Cryo-ultramicrotomy images are indicated on the left, corresponding TEM images on the right. (**A**) vs. (**B**) cells without treatment; (**C**) vs. (**D**) treatment with NCL195 at 2 µg/mL showing no effect of NCL195 treatment on cell morphology; (**E**) vs. (**F**) cells treated with colistin at 0.125 µg/mL; (**G**) vs. (**H**) cells treated colistin 0.25 at µg/mL; (**I**) vs. (**J**) group exposed to colistin 0.25 at µg/mL + NCL195 at 2 µg/mL.

On the basis of the foregoing outcomes, subsequent experiments to determine the effect of NCL195 + colistin combination were conducted on Xen14 and Xen41 at *A*_600nm_ = 0.1 followed by a 1 h drug treatment. TEM of Xen14 sections cut under cryo conditions provided a clear delineation of the membrane structure, showing the outer and inner membrane and wall peptidoglycan layer, typical of Gram-negative bacteria compared with a more traditional, resin embedded TEM preparation (Figure 7A vs. Figure 7B). This result was observed in both the untreated and NCL195-treated cells (Figure 7C vs. Figure 7D).

For Xen14 cells exposed to colistin at 0.125 µg/mL and sectioned under cryo conditions, mesosome-like structures and swollen membranes were observed, whereas conventional TEM micrographs of cells exposed to colistin at 0.125 µg/mL showed no difference in membrane morphology compared to untreated cells (Figure 7E vs. Figure 7F). With increased colistin concentration (0.25 µg/mL), swollen envelopes and mesosome-like structures (m) were observed following cryo preparation (Figure 7G) in addition to the presence of tubular appendages (t) observed using the traditional TEM technique (Figure 7H). Cells treated with a combination of NCL195 (2 µg/mL) and colistin (0.25 µg/mL) and processed under cryo conditions exhibited increased morphological damage including coronate tubular appendages and mesosome-like structures (Figure 7I). Cells treated with the combination and visualized using conventional TEM also showed increased morphological damage, coronate tubular appendages and a swollen and detached membrane (Figure 7J) similar to that observed with cells treated with 0.25 µg/mL colistin alone. These results are summarized in Table 2.

The observed morphological effects of NCL195 in the NCL195 + colistin combination are consistent with the dissipation of inner cell membrane potential demonstrated in our recent work [10], potentially resulting in leakage of vital metabolites [25].

**Figure 8:**
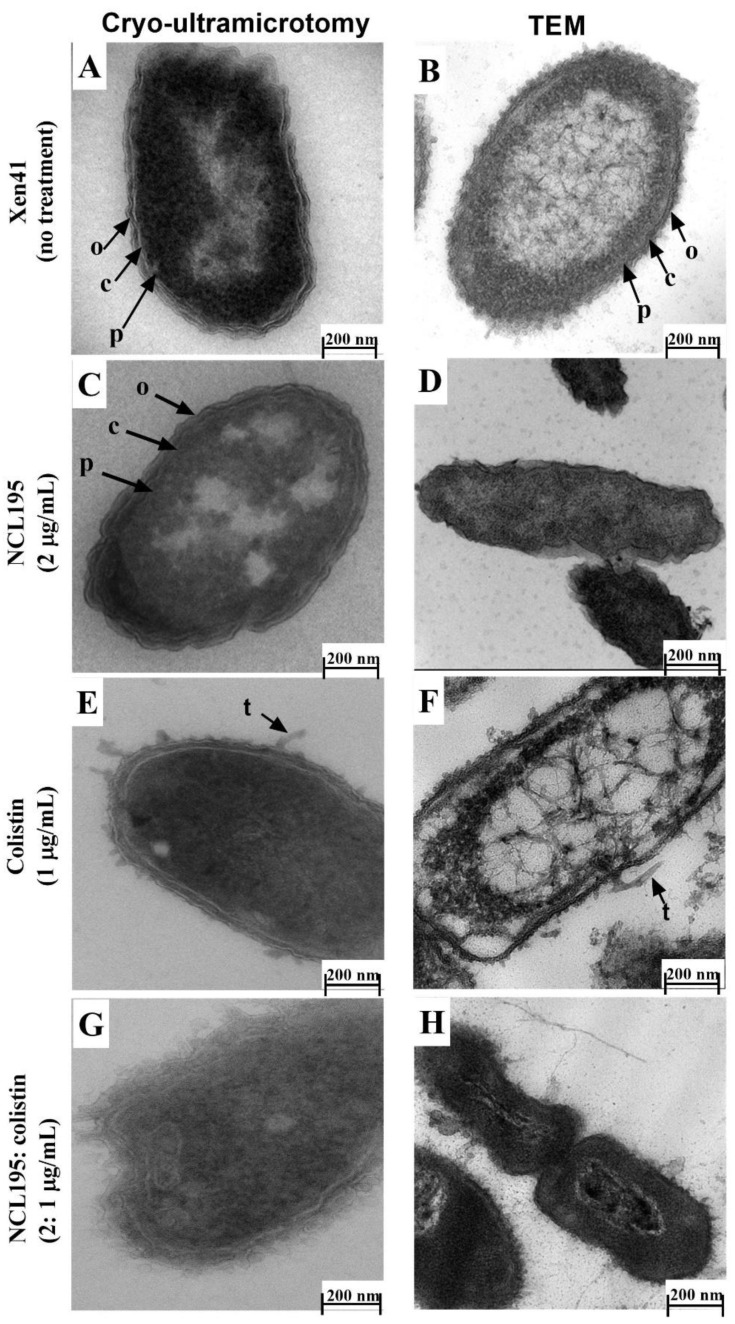
Comparison of Xen41 cells with or without antibiotic treatment and processed by Tokuyasu cryo-ultramicrotomy vs. conventional TEM. Approximately 5 × 10^8^ cells were treated for 1 h. (**A**) vs. (**B**) cells without treatment; (**C**) vs. (**D**) treatment with NCL195 at 2 µg/mL showing clearer delineation of intact outer membrane, cell wall and plasma membrane than TEM images and no effect of NCL195 treatment on cell morphology; (**E**) vs. (**F**), Xen41 cells treated with colistin at 1 µg/mL and (**G**) vs. (**H**) Xen41 cells exposed to a combination of NCL195 at 2 µg/mL and colistin at 1 µg/mL.

As seen with Xen14, untreated Xen41 cells processed under cryo conditions produced a clear image of bacterial morphology with cell walls and inner and outer membranes clearly distinguishable (Figure 8A) compared to traditional TEM processing methods (Figure 8B). Cells treated with NCL195 alone under cryo conditions showed similar ultrastructural morphology as the control cells (Figure 8C), although a slightly wavy membrane morphology was observed when using traditional TEM preparations (Figure 8D).

The addition of colistin (1 µg/mL) produced clearly visualized membrane damage in cells processed under cryo conditions (Figure 8E), whereas a ruffling of the cells and wavy cell membrane structure was observed following traditional TEM embedding (Figure 8F). Increased ultrastructural damage was observed following treatment with the combined colistin and NCL195 treatment (Figure 8G vs. Figure 8H). Again, cells processed under cryo conditions showed clearer increased morphological changes with broken outer membranes and mesosome-like structures within the cell (Figure 8G vs. Figure 8H). These results are summarized in Table 3.

## 4. Conclusions

In this study, we describe optimized TEM conditions for visualizing changes to Gram-negative bacterial morphology induced by treatment with a combination of NCL195, a novel pyrimidine, and colistin. We show that cacodylate buffer works better than PBS buffer, and that fixative containing 4.0% formaldehyde, 1.25% glutaraldehyde, CaCl_2_ 0.01 M, 4% sucrose and 0.075% ruthenium red, 0.075% L-lysine acetate is the optimal mixture for the stability of bacterial cell membrane. We also suggest using LR-White resin due to its ease of use during TEM processing. Additionally, we show the cryo-ultramicrotomy technique provides higher resolution, artifact reduction, clearer visualization of bacterial cytoskeleton and better preservation of bacterial structural integrity compared to conventional TEM processing methods. To our knowledge, this study is the first to use Tokuyasu cryo-ultramicrotomy to examine the effects of multiple drug-interactions on the bacterial cell surface. Cryo-ultramicrotomy can also be employed in conjunction with other imaging techniques such as that described for correlative light and electron microscopy [33]. We suggest cryo-ultramicrotomy can be used for a wide range of applications including host-pathogen interaction studies and high-resolution visualization of macromolecular interactions occurring on the prokaryotic surface or other biological membranes. These should promote a better understanding of complex cellular and molecular interactions.

## Figures and Tables

**Table 1:** Optimization of sample preparation for TEM and cryo-ultramicrotomy.

Method	Buffer	Fixation	Post-fixation	Resin
Procedure 1 (TEM)	Cacodylate + 4% sucrose	3.0% formaldehyde, 0.035% glutaraldehyde, 4% sucrose and 0.075% ruthenium red, 0.075% L-lysine acetate	1% osmium tetroxide; 0.075% ruthenium red	LR-White
Procedure 2 (TEM)	PBS + 4% sucrose	4.0% formaldehyde, 1.25% glutaraldehyde, 0.01 M CaCl_2_, 4% sucrose and 0.075% ruthenium red	1% osmium tetroxide; 0.075% ruthenium red;	Epon-Araldite
Procedure 3 (TEM)	Cacodylate	4.0% formaldehyde, 1.25% glutaraldehyde, 0.01 M CaCl_2_ and 0.075% ruthenium red, 0.075% L-lysine acetate	1% osmium tetroxide; 0.075% ruthenium red	Epon-Araldite
Procedure 4 (TEM)	Cacodylate + 4% sucrose	4.0% formaldehyde, 1.25% glutaraldehyde, 0.01 M CaCl_2_, 4% sucrose and 0.075% ruthenium red, 0.075% L-lysine acetate	1% osmium tetroxide; 0.075% ruthenium red	LR-White
Procedure 5 (TEM)	Cacodylate + 4% sucrose	4.0% formaldehyde, 1.25% glutaraldehyde, 0.01 M CaCl_2_, 4% sucrose and 0.075% ruthenium red, 0.075% L-lysine acetate	1% osmium tetroxide; 0.075% ruthenium red	Epon-Araldite
Procedure 6 (Cryo-ultramicrotomy)	Cacodylate + 4% sucrose	4.0% formaldehyde, 1.25% glutaraldehyde, 0.01 M CaCl_2_, 4% sucrose and 0.075% ruthenium red, 0.075% L-lysine acetate	N/A	N/A

**Table 2:** Comparison of cryo-ultramicrotomy preparation and traditional TEM methods for visualizing the effect of drug treatments on Xen14 cell membrane.

Treatment	Cryo-Ultramicrotomy	Traditional TEM
No treatment	clear delineation of outer and inner membrane, cell wall and peptidoglycan layer	poor delineation of membrane structure
NCL195 (2 µg/mL)	same as a control	same as a control
Colistin (0.125 µg/mL)	mesosome-like structures; swollen membranes	same as a control
Colistin (0.25 µg/mL)	mesosome-like structures; more swollen membranes	presence of tubular appendages
NCL195/colistin (2/0.25 µg/mL)	increased morphological damage; coronate tubular appendages; mesosome-like structures	increased morphological damage; coronate tubular appendages; swollen and detached membrane

**Table 3:** Comparison of TEM using cryo-ultramicrotomy and traditional preparation methods to visualize the effect of drug treatments on Xen41 cell membrane.

Treatment	Cryo-Ultramicrotomy	Traditional TEM
No treatment	clear delineation of outer and inner membrane, cell wall and peptidoglycan layer	poor delineation of membrane structure
NCL195 (2 µg/mL)	same as control	slightly wavy membrane morphology
Colistin (1 µg/mL)	clearly visualized membrane damage; presence of tubular appendages	ruffling of the cells; wavy cell membrane structure; presence of tubular appendages
NCL195/colistin (2/1 µg/mL)	clearer increased morphological changes; broken outer and inner membrane; mesosome-like structures	increased ultrastructural damage; damaged outer and inner membrane

## Data Availability

The data presented in this study are available on request from the corresponding author. The data are not publicly available due to size and access restrictions.
